# Role of Herpes Simplex Virus Type 1 (HSV-1) Glycoprotein K (gK) Pathogenic CD8^+^ T Cells in Exacerbation of Eye Disease

**DOI:** 10.3389/fimmu.2018.02895

**Published:** 2018-12-07

**Authors:** Ujjaldeep Jaggi, Shaohui Wang, Kati Tormanen, Harry Matundan, Alexander V. Ljubimov, Homayon Ghiasi

**Affiliations:** ^1^Department of Surgery, Center for Neurobiology and Vaccine Development, Cedars-Sinai Medical Center, Los Angeles, CA, United States; ^2^Eye Program, Cedars-Sinai Medical Center, and David Geffen School of Medicine, Board of Governors Regenerative Medicine Institute, University of California, Los Angeles, Los Angeles, CA, United States

**Keywords:** ocular, eye disease, virus replication, corneal scarring, peptide, SPP, GODZ

## Abstract

HSV-1-induced corneal scarring (CS), also broadly referred to as Herpes Stromal Keratitis (HSK), is the leading cause of infectious blindness in developed countries. It is well-established that HSK is in fact an immunopathological disease. The contribution of the potentially harmful T cell effectors that lead to CS remains an area of intense study. Although the HSV-1 gene(s) involved in eye disease is not yet known, we have demonstrated that gK, which is one of the 12 known HSV-1 glycoproteins, has a crucial role in CS. Immunization of HSV-1 infected mice with gK, but not with any other known HSV-1 glycoprotein, significantly exacerbates CS, and dermatitis. The gK-induced eye disease occurs independently of the strain of the virus or mouse. HSV-1 mutants that lack gK are unable to efficiently infect and establish latency in neurons. HSV-1 recombinant viruses expressing two additional copies of the gK (total of three gK genes) exacerbated CS as compared with wild type HSV-1 strain McKrae that contains one copy of gK. Furthermore, we have shown that an 8mer (ITAYGLVL) within the signal sequence of gK enhanced CS in ocularly infected BALB/c mice, C57BL/6 mice, and NZW rabbits. In HSV-infected “humanized” HLA-A^*^0201 transgenic mice, this gK 8mer induced strong IFN-γ-producing cytotoxic CD8^+^ T cell responses. gK induced CS is dependent on gK binding to signal peptide peptidase (SPP). gK also binds to HSV-1 UL20, while UL20 binds GODZ (DHHC3) and these quadruple interactions are required for gK induced pathology. Thus, potential therapies might include blocking of gK-SPP, gK-UL20, UL20-GODZ interactions, or a combination of these strategies.

## Role of HSV-1 Glycoproteins in Protection and Disease

HSV-1 encodes at least 85 genes (1) and 12 of these genes code for glycoproteins (1–6). These glycoproteins (gB, gC, gD, gE, gG, gH, gI, gJ, gK, gL, gM, and gN) are the major inducers and targets of humoral and cell-mediated immune responses following infection (4, 7–10). We have constructed recombinant baculoviruses expressing high levels of each of the 10 HSV-1 glycoprotein genes (3–6, 11–20). Based on immunization studies in mice, we have classified these 10 baculovirus-expressed genes into four groups: (i) Immunization with gB, gC, gD, gE, or gI completely protects mice against lethal challenge (11–15); (ii) No significant protection was seen with gH, gJ, and gL (5, 6, 16–18); (iii) Immunization with gK leads to severe exacerbation of eye disease (3, 19, 20); and (iv) Immunization with gG also showed a tendency to be harmful (6, 16).

## Herpes Stromal Keratitis (HSK)

HSV-1-induced CS, also broadly referred to as herpes stromal keratitis (HSK), can lead to blindness. HSV-1 is the leading cause of corneal blindness due to an infectious agent in developed countries (21–26). In the U.S., ~30,000 people suffer recurrent ocular HSV episodes annually, requiring doctor visits, medication, and in severe cases, corneal transplants. It is estimated that 70–90% of American adults have antibodies to HSV-1 and/or HSV-2, and about 25% of these individuals have clinical symptoms upon routine clinical exam (21–26). HSV-1 is responsible for >90% of ocular HSV infections. The global incidence of Herpes Keratitis is roughly around 1.5 million including 40,000 new cases of severe visual impairment and blindness each year (27). A significant proportion (15–50%) of primary genital herpes is caused by HSV-1, and recent studies indicate that the proportion of first clinical episode genital herpes due to HSV-1 is increasing (28–30). Despite the frequent recurrence of ocular herpes, there are no vaccines available for HSV infections (31). In addition, no drug has been FDA approved for the prevention of ocular recurrences.

## Herpes Infection as an Immune-mediated Event

Viral infections trigger the host immune response in a way that the immune system gets highly compromised (32). Chronic viral infections have evolved different mechanisms by which they escape the response of protective immune response presenting a serious challenge to the infected host (32). Many factors come into play, which are responsible for causing the spread of the disease and, if not properly managed, it can pose a serious threat to the host (33). Current therapies for treatment of ocular HSV-1 infection include the use of antiviral drugs and corticosteroids which can minimize the lesions but often lead to certain side effects (34); therefore, new measures need to be adopted. Studies on mouse models of ocular HSV-1 infection have unraveled many insights into the disease pathogenesis paving ways to future innovative therapies (35, 36). It is well-documented that HSV-1 pathology is a consequence of the immune response mounted by the host after virus infection and therefore, it is considered an immunopathological disease (37). During the course of HSV-1 infection, a series of events take place involving the replication of virus in the epithelial cells and formation of new blood vessels which accounts for the angiogenic response (33). The infectious virus is cleared from the eye by day 6–7 post infection, but secondary effects lead to the induction of strong cellular immune response with the appearance of immune cell infiltrates in the cornea resulting in damage to the eye (38, 39). Recent studies done in mice showed that HSV-1 infection also leads to corneal nerve damage/retraction, which results in loss of corneal sensitivity and blink reflexes and promotes HSK pathogenesis (40).

In this review, we will discuss the role of gK in HSV-1-induced CS and will propose new potential therapeutic approaches to reduce or control gK-induced CS.

## gK and its Role in Herpes Infection

gK encoded by the UL53 gene is one of the HSV-1 glycoproteins and is expressed on the virions (1, 3, 41). gK is a highly hydrophobic 338-amino acid protein with a predicted molecular mass of 37 kDa (1). gK has a cleavable 30-amino-acid NH2-terminal signal sequence and is N-glycosylated on amino acids 48 and 58 (1, 42, 43). In HSV-1 infected cells, gK is expressed as a 39 kDa high-mannose precursor polypeptide, designated precursor gK (pgK), which is further glycosylated to produce a 41 kDa mature glycoprotein (41). When we expressed gK using a recombinant baculovirus, four gK-related baculovirus-expressed polypeptides of 29-, 35-, 38-, and 40-kDa were detected (3). The 35-, 38-, and 40-kDa species were susceptible to tunicamycin treatment revealing that they were N-glycosylated. The 35-kDa protein represented the cleaved and partially glycosylated peptide, whereas the 29-kDa protein represented the cleaved unglycosylated peptide. gK translated *in vitro* had a molecular mass of 36 kDa with four possible membrane-spanning regions (43, 44). Studies using insertion/deletion mutants have shown the importance of gK in virion morphogenesis and egress (45–47). Deletion of gK results in the formation of extremely rare microscopic plaques indicating that gK is required for virus replication, a concept that is supported by the observation that gK-deficient virus can only be propagated on complementing cells that express gK (45, 46).

**Figure 1:**
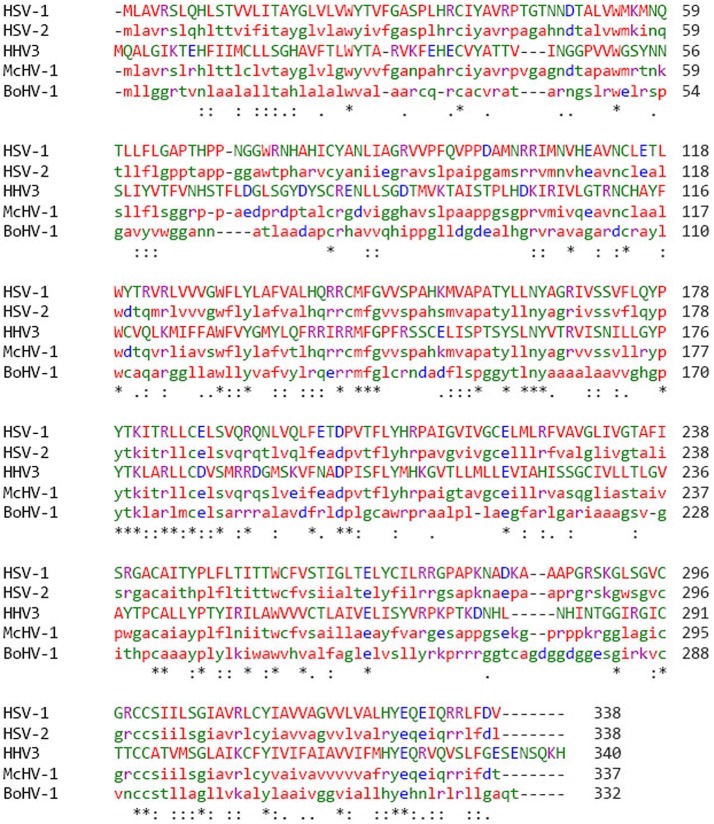
gK protein sequence alignment in different strains of alphaherpes viruses. Protein sequence was aligned by clustal omega and percentage of amino acid homology was compared among different groups of Herpes viruses. HSV-1 has 85% homology with HSV-2, 66% homology with McHV-1, 34% homology with BoHV-1, and 28% homology with VZV. Stars (^*^) indicate that the amino acids sequences are the same.

gK shares 100% amino acid homology between different strains of HSV-1 (1, 48, 49). Similar to HSV-1 gK, HSV-2 is also 338 amino acids long but with ~84% amino acid homology (1, 50, 51). In addition to HSV-1 and HSV-2, gK is also present in other members of alphaherpes viruses. The gK homologies between different alphaherpes viruses are shown in Figure 1. Protein sequence alignment is illustrated using clustal omega, in which we show that gK from Macacine Herpes Virus 1 (McHV-1), Bovine Herpes Virus 1 (BoHV-1) and Varicella zoster virus (HH3, VZV), share 66, 33 and 28% sequence homology with HSV-1 gK, respectively (Figure 1). Kousoulas' group reported that HSV-1 gK is a structural component of virion particles and demonstrated that gK is a Golgi complex-dependent glycosylated species (52). Previously, it was shown that HSV-1 UL20 is required to interact with gK for HSV-1 infection (53). Also, a similar study with Bovine herpes virus type 1 (BoHV-1), a member of the alphaherpes virus family, demonstrated that BoHV-1 gK and UL20 proteins function together in a manner similar to HSV-1 gK and UL20 in virus spread and infection. UL20 has a role in cell surface expression of gK but is not required for gK-mediated cell fusion (54). It has also been demonstrated that UL20 plays a critical role in virion envelopment, and virions lacking either gK or UL20 fail to form an envelope. A similar role has been assigned to HSV-1 UL37 protein in cytoplasmic virion envelopment, and it was shown that UL37 interacts with gK-UL20 protein complex in infected cells and facilitates in virion cytoplasmic envelope (55).

Recently, we reported that HSV-1 UL20 binds to and is palmitoylated by GODZ (also known as DHHC3), a Golgi apparatus-specific Asp-His-His-Cys (DHHC) zinc finger protein and an essential component of virus infectivity (56). Palmitoylation of UL20 is critical for gK cell surface localization. Thus, the use of GODZ dominant-negative mutant or GODZ shRNA can be a potential way of inhibiting the binding of UL20 to GODZ, which can affect gK localization and viral replication. We further showed the importance of GODZ in HSV-1 infection using knockout mice. GODZ^−/−^ mice ocularly infected with HSV-1 had reduced ocular virus replication and reduced latency-reactivation as compared with wild type control mice. Our study also showed that the absence of GODZ resulted in blocking of palmitoylation of UL20 and affected the localization of gK along with the reduced expression levels of UL20, gK, and gB transcripts in the corneas of HSV-1 infected GODZ^−/−^ mice (57).

Recently, it was shown that intramuscular injection with HSV-1 (F) mutant virus, which lacks the expression of gK conferred significant protection against either virulent HSV-1 strain McKrae or HSV-2 strain G intravaginal challenge in mice (58). To test if disruption of gK/UL20 interactions with gB would lead to reduced viral load, a recombinant virus (VC2) was constructed with specific mutations in gK and its binding protein UL20. Intramuscular injection with VC2 indeed protected 100% of mice against virulent HSV-1 strain McKrae or HSV-2 strain G challenges by providing cross-reactive humoral and cellular immunity (59).

**Figure 2:**
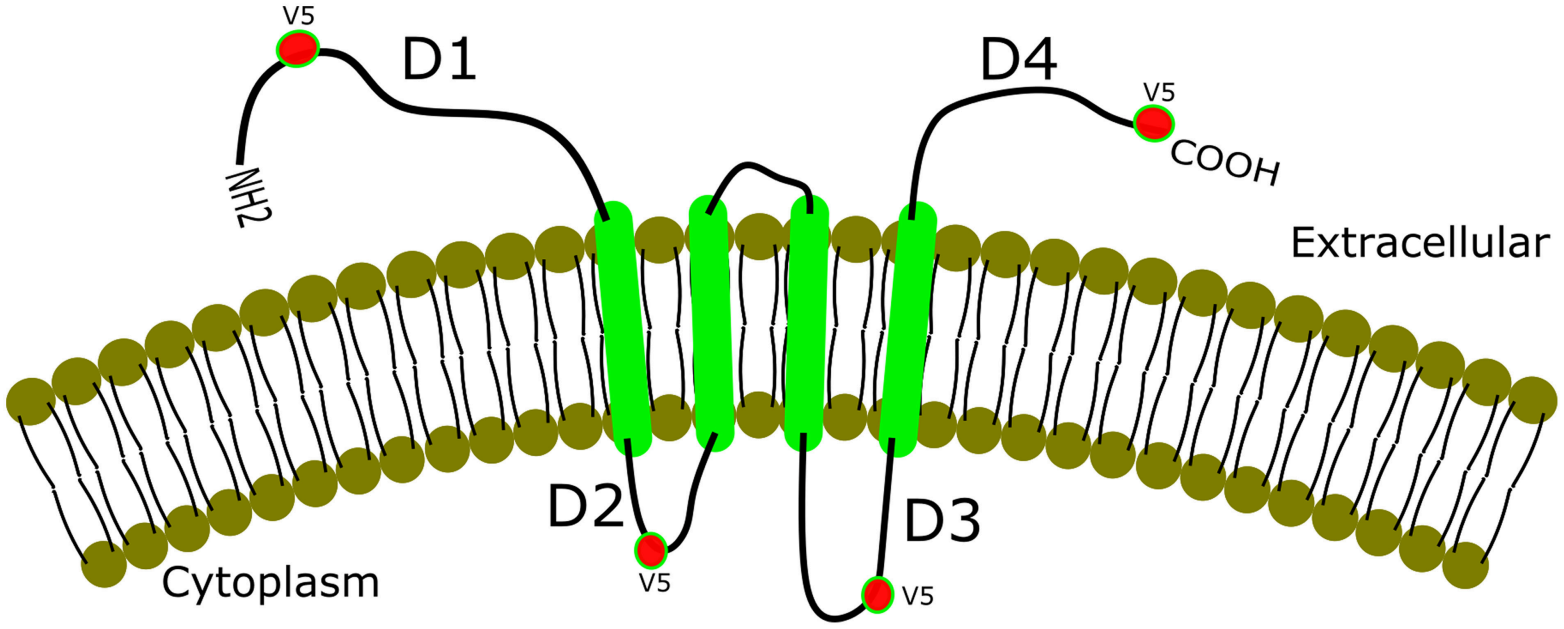
Co-localization of gK and SPP. gK is a highly hydrophobic protein with four transmembrane domains. Epitope-tagging of four different domains of gK is shown with a strong co-localization of the two cytoplasmic domains (labeled D2 and D3 in the figure). Extracellular domains (D1 and D4), on the other hand, show weak or no co-localization with SPP in RS, HeLa, and Vero cell lines.

Additionally, gK binds with different affinity in different cell types (Figure 2) to signal peptide peptidase (SPP) also known as minor histocompatibility antigen H13 (60). To illustrate this binding, recombinant gKV5DI, gKV5DII, gKV5DIII, and gKV5DIV viruses were constructed expressing V5 epitope tags in frame within domains I, II, III, and IV of gK, respectively (52, 61). We infected rabbit skin (RS), HeLa and Vero cells with each virus and evaluated the co-localization of V5-gK and endogenous SPP. There was a strong co-localization in all the cell lines (RS, HeLa and Vero) when the V5 tag was expressed on cytoplasmic domains (II and III) compared to when it was expressed on extracellular domain (I and IV) (Figure 2). Binding of gK to SPP can be blocked by SPP inhibitors like aspirin, ibuprofen, L685, 458, (Z-LL)_2_ ketone, and DAPT (62). These inhibitors significantly reduced viral replication in HSV-1 infected eye and reduced pathology. Thus, blocking the binding of SPP to gK can be one of the potential approaches toward treating HSV-1 induced CS (62).

## gK and Virus Entry

HSV-1 induced CS begins with the binding of viral glycoproteins to the host cell entry receptors. There are at least seven known receptors including herpes virus entry mediator (HVEM) as well as nectin-1, nectin-2, 3-O-sulfated heparan sulfate (3-OS-HS), paired immunoglobulin-like type 2 receptor (PILRα), non-muscle myosin heavy chain IIA (NMHC-IIA), and myelin-associated glycoprotein (MAG) (2, 63–72). For gK to potentiate its disease severity, the amino terminal of gK binds to the amino terminal of gB, which leads to the virus entry and disease progression (73). gB binds to Akt-1 during virus entry and it induces Akt phosphorylation and intracellular calcium release. A recent study done by Kousoulas' group showed that deletion of amino acids 31–68 within the amino terminus of gK inhibits gB binding to Akt-1 and thus blocks virus entry and its progression (74). Studies by the same group showed that both gK and PlLRα (paired immunoglobulin–like type 2 receptor α) bound gB in infected cells and that the association between gB-PILRα protein complex regulates membrane fusion of virus and the host cell which aids in virus penetration (75). Along with the role of amino terminus of gK in virus entry, a recent study described the role of two conserved N-linked glycosylation sites (N48 and N58) of gK in virus-induced cell fusion and replication (76). Mutation at N58 to alanine (N58A) resulted in extensive virus-induced cell fusion. The same group showed that mutation of cysteine residues within the amino terminus of gK, C37, and C114, led to significant reduction in virus production (76). In addition, gK plays a vital part in the recruitment of other viral glycoproteins into intracellular virus assembly. A recent study found that gM plays a major role in synergy with gK/UL20 in the incorporation of gD and gH/gL into mature virions (74).

## Role of gK-induced Cellular Responses

Adaptive immune responses play a major role in HSV-1 pathogenesis. The role of CD8^+^ T cells in HSV-1 pathogenesis is currently unclear and needs deeper investigation. There are studies reporting that CD8^+^ T cells play a protective role, whereas other studies show that CD8^+^ T cells exacerbate the disease pathogenesis (77, 78). There is evidence supporting that gK is the only HSV-1 glycoprotein responsible for exacerbation of HSV-1 induced corneal scarring (CS). Research done by our team shows that a virus construct of HSV-gK^3^ which is derived from the virulent HSV-1 strain McKrae mediates critical effects on HSV-1 pathogenicity in mice (79). Mice infected with HSV-gK^3^ showed severe CS compared with control mice infected with wild type virus. HSV-gK^3^ infected mice had elevated levels of virus replication and also had significantly higher number of CD8^+^ T cells (79). Depletion of CD8^+^ T cells and not CD4^+^ T cells reduced CS in HSV-gK^3^ infected mice to the level of wild type infected mice. Overall, we have shown that exacerbation of eye disease in response to gK immunization or following ocular infection with recombinant viruses expressing additional copies of gK is associated with CD8^+^T cell and not CD4^+^T cell responses. Other studies have shown that CD4^+^ T cells are involved in HSK (80–83). Thus, in the context of CD8^+^-induced gK pathogenicity the role of CD4^+^ T cells to disease or protection cannot be ruled out.

We previously looked into what region of gK participates in T cell proliferation and subsequently IFN-γ production (84). To this end, a panel of 33 overlapping peptides spanning all 338 amino acids of the gK polypeptide were produced. Splenocytes from mice were stimulated with each peptide individually both *in vivo* and *in vitro*. We found that out of 33 peptides, peptide 2 was involved in T cell proliferation and IFN-γ production *in vivo* and *in vitro* and accounted for 52% of CTL activity *in vivo*. The percentages of IFN-γ production by both CD4^+^ T and CD8^+^T cells *in vivo* and the CTL responses are illustrated in Table 1. *In vitro* results showed that CD8^+^ T cells produced more IFN-γ compared to CD4^+^ T cells. Our study confirmed that both CD4^+^ and CD8^+^T cells produced IFN-γ when stimulated with peptide 2, but IFN-γ production by CD4^+^ T was CD8^+^ T cell-dependent. In connection with our mapping studies (3, 79), we identified a highly conserved gK epitope (ITAYGLVL) within the peptide STVVLITAYGLVLVW, which served as an immunodominant gK T cell stimulatory region both *in vitro* and *in vivo* (84). This peptide is highly conserved between HSV-1 and HSV-2 strains. To investigate its role in HSV-1 infection, the octamer (8mer) was administered as an eye drop an hour before ocular infection. This resulted in a significant increase in viral replication leading to enhancement of CS, along with strong cytotoxic CD8^+^ T cell responses and IFN-γ production (85). Mutations in the signal sequence of gK using recombinant viruses that expressed two additional copies of the mutated (MgK) or native (NgK) form of the gK blocked cell surface expression of gK in RS cells resulting in reduced reactivation and hence, less ocular disease when compared to RgK (revertant) virus. This study confirms the role of octamer within the signal sequence of gK in HSV-1 pathogenesis (86). Another study showed that the amino terminus of gK was essential for neuroinvasiveness and acute HSK using a recombinant HSV-1 (McKΔgK31-68), which was lacking the 38 amino acids from gK amino terminus. In McKΔgK31-68 mutant viral infection, there were no significant disease symptoms (87).

**Table 1:** IFN-γ production and CTL activity from both CD4^+^T and CD8^+^T cells when stimulated with gK synthetic peptides^a^.

**Peptide**	**gK aa**	**CD4^**+**^**IFN**γ^+^**	**CD8^**+**^**IFN**γ^+^**	**CTL activity**
1	MLAVRSLQHLSTVVL	2%	1%	9%
2	STVVLITAYGLVLVW	21%	8%	52%
3	LVLVWYTVFGASPLH	3%	2%	–
4	ASPLHRCIYAVRPTG	ND	ND	ND
5	VRPTGTNNDTALVWM	ND	ND	ND
6	ALVWMKMNQTLLFLG	ND	ND	ND
7	LLFLGAPTHPPNGGW	ND	ND	ND
8	PNGGWRNHAHICYAN	ND	ND	ND
9	ICYANLIAGRVVPFQ	ND	ND	ND
10	VVPFQVPPDAMNRRI	ND	ND	ND
11	MNRRIMNVHEAVNCL	ND	ND	ND
12	AVNCLETLWYTRVRL	ND	ND	ND
13	TRVRLVVVGWFLYLA	ND	ND	ND
14	FLYLAFVALHQRRCM	ND	ND	ND
15	QRRCMFGVVSPAHKM	ND	ND	ND
16	PAHKMVAPATYLLNY	ND	ND	ND
17	YLLNYAGRIVSSVFL	ND	ND	ND
18	SSVFLQYPYTKITRL	ND	ND	ND
19	KITRLLCELSVQRQN	ND	ND	ND
20	VQRQNLVQLFETDPV	ND	ND	ND
21	ETDPVTFLYHRPAIG	ND	ND	ND
22	RPAIGVIVGCELMLR	ND	ND	ND
23	ELMLRFVAVGLIVGT	ND	ND	ND
24	LIVGTAFISRGACAI	ND	ND	ND
25	GACAITYPLFLTITT	ND	ND	ND
26	LTITTWCFVSTIGLT	ND	ND	ND
27	TIGLTELYCILRRGP	ND	ND	ND
28	LRRGPAPKNADKAAA	ND	ND	ND
29	DKAAAPGRSKGLSGV	ND	ND	ND
30	GLSGVCGRCCSIILS	ND	ND	ND
31	SIILSGIAVRLCYIA	ND	ND	ND
32	LCYIAVVAGVVLVAL	ND	ND	ND
33	VLVALHYEQEIQRRL	ND	ND	ND

a *Splenocytes from naive BALB/c mice were prepared and tested for in vivo cytolytic activity as was reported (84). Cells were pulsed with respective peptides for 18 h and cytolytic activity was measured by FACS analysis. ND, Not detected*.

Hendricks's group looked at HSV-1-specific CD8^+^ T cell repertoire in C57BL/6 mice that respond to 376 predicted HSV-1 CD8^+^ T cell epitopes in C57BL/6 mice (88). Out of 376 HSV-1 CD8^+^ T cell epitopes, only 19 (gB_498−505_ and 18 subdominant epitopes) stimulated CD8^+^T cells in spleen and TG of HSV-1 infected mice. The data in comparison to all these epitopes demonstrated that majority of the CD8^+^T cells in spleen and TG of HSV-1 infected mice responded to gB_498−505_ HSV-1 epitope and as expected the authors showed that gK peptide corresponding to aa 54–62 was recognized by CD8^+^ effector T cells in TG and spleen of infected mice (88). So, collectively our study and Hendricks's group showed that CD8^+^ T cells in C57BL/6 mice recognize various HSV-1 epitopes, especially gK but this is in contrast to human TG study in which it was indicated that the human TG is an immunocompetent environment for both CD4^+^ and CD8^+^ T cell recognition of diverse HSV-1 proteins expressed during latent infection (89). The infiltration of CD4^+^ and CD8^+^ T cells was measured in 15 TG of eight HSV-1 IgG seropositive donors by flow cytometry. It was found that there were equivalent numbers of CD4^+^ and CD8^+^ T cells, with a median ratio of CD4^+^ and CD8^+^ T cells of 0.99 (range 0.01–9.32). Also, peptide-specific CD8^+^ T cell responses were detected in two TG which recognized four HLA-A^*^0101-restricted peptides: gL_66−74_, gK_201−209_ and two VP16 peptides, VP16_90−99_, and VP16_479−488_. It was concluded that human intra-TG HSV-1-specific CD8^+^ T cell responses were directed to a relatively restricted number of viral proteins in each person (89). CD8^+^ T cell depletion in gK immunized mice resulted in reduced severity of gK-induced CS in mice infected with wild type HSV-1 strain McKrae (90). The underlying mechanism of CD8^+^ T cell pathology in HSV-1 infected gK-immunized mice was confirmed by the presence of CD8^+^CD25^+^ regulatory T cells in cornea of gK immunized mice (78). Thus, similar to our results, the published studies confirmed our finding that gK induces CD8^+^ T cell responses and this response is contributing to enhancement of eye disease. This is probably the reason why depletion of CD8^+^ T cell but not CD4^+^ T cells reduced gK exacerbation of eye disease (79).

Previous studies revealed that gK sera caused antibody-dependent enhancement (ADE) of HSV-1 infection, which may explain the higher viral load in the corneas of gK-vaccinated mice (91). ADE differs from the usual process of virus entry where virus enters the host cells by binding of the viral glycoproteins to the cellular receptors. In ADE, IgG binds to a virus allowing the virus-antibody complex to attach to the host cells containing Fc receptors. A comparative study between HSK sera and non-HSK sera indicated that about 75% of found neutralizing antibodies were associated with gB, gC, gD, gE, and gI. It was shown by ELISA that sera from HSK group had significantly higher anti-gD and anti-gK antibodies than sera from non-HSK group. Similarly, when mice were immunized with gD+gK, levels of neutralizing antibody titers in immunized mice were reduced by ~30% in comparison to mice immunized with gD alone. This is in agreement with data showing that mice immunized with gD showed T_H_1 response whereas mice immunized with gK exhibited a T_H_1 + T_H_2 response. T_H_1 + T_H_2 response in gK-immunized mice enhances the eye pathology (79).

## Role of gK in HSV-1 Chronic Infection

One of the hallmarks of HSV-1 infection is the ability of the virus to establish latency in sensory neurons of an infected host (92–96). In neurons, expression of more than 80 genes of HSV-1 that occurs during lytic infection is drastically modified. The latency-associated transcript (LAT) is the only gene product consistently detected in abundance during latency in infected mice, rabbits, and humans (92–94, 97, 98). In mice, spontaneous reactivation occurs at extremely low levels and infectious virus is rarely detected. When mouse TGs are removed at autopsy and explant co-cultivated in tissue culture with indicator cells, latent virus reactivates and can be observed by the detection of cytopathic effects (CPE) on the indicator cell monolayer. Reactivation from latency is not immediate, and typically CPE is not detected during the first 2–3 days of explant co-cultivation. In contrast, when cell-free lysates of latently infected TG are plated on indicator cells, CPE is not seen (20, 99). This indicates that there was no infectious virus present in the TGs and confirms that reactivation from latency by co-cultivation requires explant of intact neurons (100, 101). We previously reported that vaccination of BALB/c mice with the baculovirus-expressed gK or passive transfer of anti-gK purified IgG to naïve BALB/c mice causes severe exacerbation of HSV-1 induced CS following ocular challenge (3, 19). In addition, a productive chronic infection, rather than a latent infection, is found in most TGs (20). Similar to gK immunization or anti-gK IgG transfer, ocular challenge of naive ${\text{A}}_{\text{β}}^{-/-}$ but not β_2_m^−/−^ mice with HSV-1 did not result in chronic infections. Surprisingly, however, when ${\text{A}}_{\text{β}}^{\text{O}/\text{O}}$ mice were vaccinated even with media alone or adjuvant alone prior to ocular challenge, a chronic, rather than a latent, infection was seen (102). When SCID mice which lack both T and B cells, are challenged ocularly with HSV-1, the surviving mice have a chronic, rather than a latent infection in their TG, with significant amounts of infectious virus (103). Thus, gK enhancement of eye disease may be associated with suppression of a certain protective arm of immune response, while enhancing the harmful arm.

From the studies done above, we can make an observation that both gK and LAT plays an important role in pathogenesis of CS. Where LAT is directly involved in reactivation of the virus which leads to pathogenicity, gK follows an indirect approach toward pathology by binding to SPP, which is known to cause virus infectivity and activation of CD8^+^T cells which in turn produce high amounts of IFN-γ and cytotoxic effects. We have also studied that deletion of gK in neural cell cultures leads to inhibition of virus to undergo transport in anterograde or retrograde directions, in short inhibiting the reactivation of virus. gK is known to cause severe immunopathology including cornea scaring, its effect on nerve damage can be detrimental to the host. A recent report shows that deletion of gK can significantly attenuate nerve damage caused by HSV-1 infection (104).

## Possible Use of gK for Control of HSV-1 Induced CS

Many steps have been evaluated in resolving the lesions caused after HSV-1 infection such as administering anti-viral drugs and using corticosteroids, which provide limited control of viral replication and are also known to cause side effects (105). Drugs like trifluridine and ganciclovir are being extensively used for patients with HSV-1 infection along with topical acyclovir to control active viral replication (106). Therefore, we need effective measures to control virus reactivation. It would be more clinically beneficial if new means are developed to prevent the initiation of pathogenesis. As discussed above, HSV-1 gK binds to SPP and UL20, while UL20 binds to GODZ (56, 60). Therefore, blocking the binding of gK to SPP, gK to UL20, or UL20 to GODZ or their combinations could be used to block HSV infectivity and pathogenesis. For example, previously we have shown that blocking the binding of gK to SPP by using SPP inhibitors can reduce CS in infected mice.

## Conclusions

**Figure 3:**
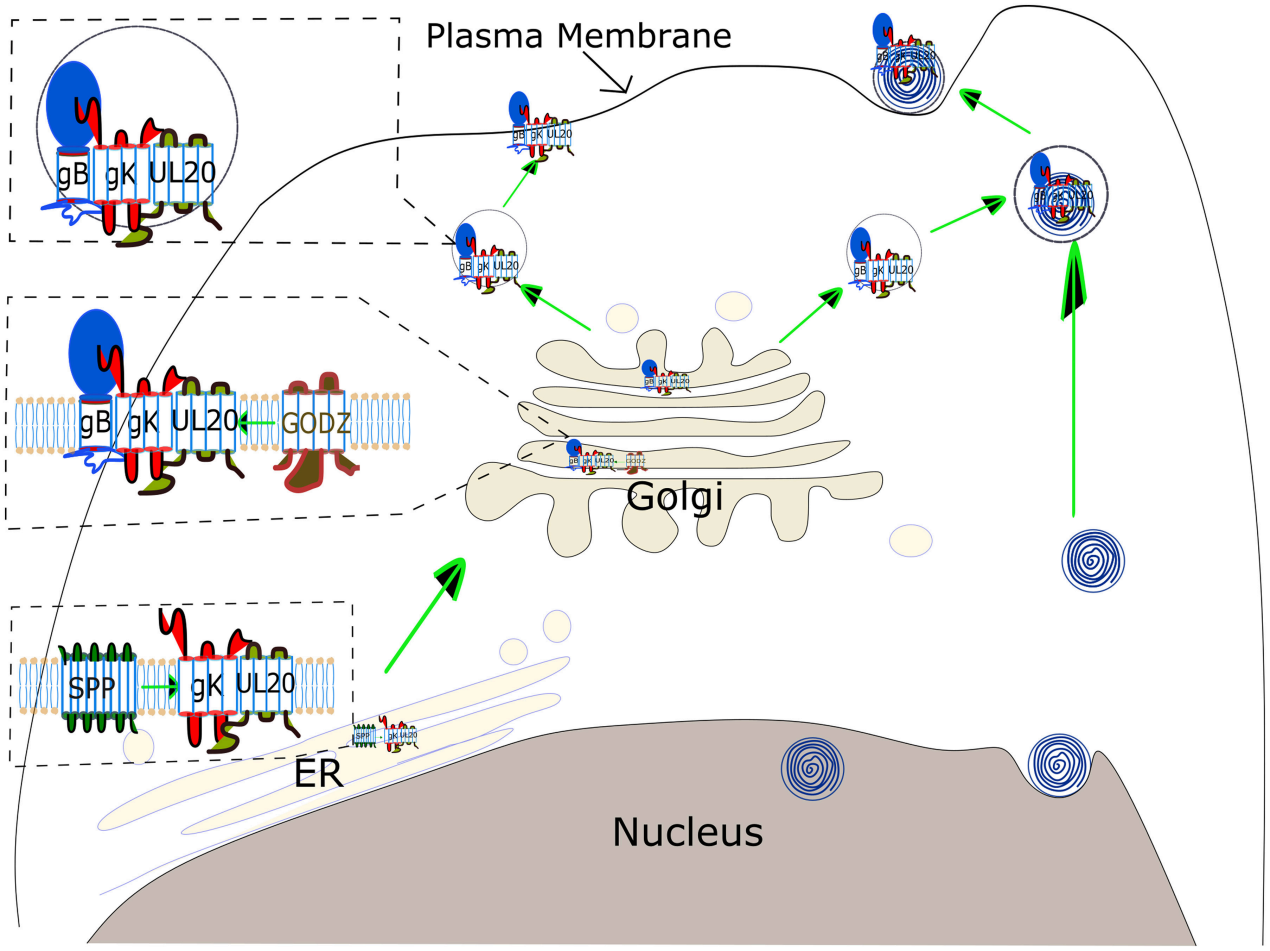
Schematic view of gK transportation and its role in virus egress. gK binds to SPP in the ER, which is necessary for virus replication, although the precise binding domain between these two proteins has not been identified yet. gK has a signal sequence in its N-terminus, however, it is not clear if this gK signal peptide is cleaved by SPP. gK is then transported to the Golgi via a UL20-dependent pathway. UL20 is palmitoylated by the host *cis*-Golgi protein GODZ, and this post-translational modification by GODZ is necessary for transport of the gK, and UL20 complex to the plasma membrane and virus infectivity. The gK and UL20 complex is also required for gB transportation to the cell surface. The complex of three proteins, gB, gK, and UL20, is either assembled into virus capsid emerging from the nucleus in a vesicle derived from TGN (**Upper right**) or transported directly to the plasma membrane (**Upper left**).

The journey of combating HSV-1 induced CS has started long ago, although many areas of the path of virus pathogenesis still remain unexplored (107). Seroprevalence studies have illustrated that the majority of individuals in the United States are infected with HSV-1 (108). This review focused on the role of HSV gK in the progression of disease severity. Published studies have clearly demonstrated the participation of gK in the exacerbation of CS and the immune response to gK in this process as a major pathogenic mechanism. A model of gK activity is illustrated in Figure 3. gK interacts with SPP in the endoplasmic reticulum (ER), and this interaction may be necessary for transport of gK from the ER to the Golgi. In the Golgi, gK interacts with gB and UL20. Palmitoylation of UL20 by GODZ either facilitates transport of the gB- gK- UL20 complex to the plasma membrane or viral packaging (Figure 3). Research is in progress to inhibit the function of gK in causing HSV-1 induced CS but further studies are required. Clearly, an exciting approach would be inhibiting the binding of gK to SPP, gK to UL20, and UL20 interactions with GODZ supports the goal of controlling HSK pathogenesis.

## Author Contributions

UJ, KT, and HG writing and editing. SW, HM, and AL editing. SW and KT designing figures.

### Conflict of Interest Statement

The authors declare that the research was conducted in the absence of any commercial or financial relationships that could be construed as a potential conflict of interest.
